# Epigenome-Wide Association Study of Infant Feeding and DNA Methylation in Infancy and Childhood in a Population at Increased Risk for Type 1 Diabetes

**DOI:** 10.3390/nu13114057

**Published:** 2021-11-13

**Authors:** Elizabeth Walker-Short, Teresa Buckner, Timothy Vigers, Patrick Carry, Lauren A. Vanderlinden, Fran Dong, Randi K. Johnson, Ivana V. Yang, Katerina Kechris, Marian Rewers, Jill M. Norris

**Affiliations:** 1 Department of Epidemiology, Colorado School of Public Health, Aurora, CO 80045, USA; elizabeth.walker-short@cuanschutz.edu (E.W.-S.); teresa.buckner@cuanschutz.edu (T.B.); patrick.carry@cuanschutz.edu (P.C.); LAUREN.VANDERLINDEN@CUANSCHUTZ.EDU (L.A.V.); randi.johnson@cuanschutz.edu (R.K.J.); ivana.yang@cuanschutz.edu (I.V.Y.); 2 Department of Biostatistics and Informatics, Colorado School of Public Health, Aurora, CO 80045, USA; timothy.vigers@cuanschutz.edu (T.V.); katerina.kechris@cuanschutz.edu (K.K.); 3 Barbara Davis Center for Diabetes, School of Medicine, University of Colorado Anschutz Medical Campus, Aurora, CO 80045, USA; fran.dong@cuanschutz.edu (F.D.); marian.rewers@cuanschutz.edu (M.R.); 4 Division of Biomedical Informatics and Personalized Medicine, Department of Medicine, University of Colorado Anschutz Medical Campus, Aurora, CO 80045, USA

**Keywords:** methylation, breastfeeding, infant diet

## Abstract

We assessed associations between infant diet (e.g., breastfeeding and introduction to solid foods) and DNA methylation in infancy and childhood. We measured DNA methylation in peripheral blood collected in infancy (9–15 months of age) in 243 children; and in a subset of 50 children, we also measured methylation in childhood (6–9 years of age) to examine persistence, and at birth (in cord blood) to examine temporality. We performed multivariable linear regression of infant diet on the outcome of methylation using epigenome-wide and candidate site approaches. We identified six novel CpG sites associated with breastfeeding duration using an EWAS approach. One differentially methylated site presented directionally consistent associations with breastfeeding (cg00574958, *CPT1A*) in infancy and childhood but not at birth. Two differentially methylated sites in infancy (cg19693031, *TXNIP*; cg23307264, *KHSRP*) were associated with breastfeeding and were not present at birth; however, these associations did not persist into childhood. Associations between infant diet and methylation in infancy at three sites (cg22369607, *AP001525.1*; cg2409200, *TBCD*; cg27173510, *PGBD5*) were also present at birth, suggesting the influence of exposures other than infant diet. Infant diet exposures are associated with persistent methylation differences in *CPT1A*, which may be one mechanism behind infant diet’s long-term health effects.

## 1. Introduction

Breastfeeding and complementary feeding are important early exposures that may have important long-term effects. Longer breastfeeding duration has been associated with decreased risk of obesity [1], type 2 diabetes (T2D) [2] and type 1 diabetes (T1D) [3]. It is also recommended to introduce solid foods by 6 months to avoid nutrient deficiencies, such as iron, protein, and vitamins [4]. Studies have shown that the timing of exposure to gluten in infancy is associated with the risk of celiac disease [5,6] although not consistently [7,8]. Earlier introduction to fruits and berries, root vegetables, and cereals is associated with T1D autoimmunity and T1D [9,10,11,12].

The mechanisms through which early dietary factors impact long-term health outcomes are still unknown, but could be through epigenetics, such as changes in DNA methylation [13]. DNA methylation utilizes DNA methyltransferase to methylate, or add a methyl group to, CpG (cytosine-phosphate-guanine) sites in DNA [14] to regulate gene expression. Several studies have examined the association between breastfeeding and DNA methylation in the child [15]. Total breastfeeding duration was associated with DNA methylation of the Leptin (*LEP*) and retinoid X receptor alpha (*RXRA)* genes in 1-year-old infants [16]. Moreover, the total and exclusive breastfeeding durations were associated with DNA methylation of *LEP* gene in 10-year-old children [17]. Finally, breastfeeding duration >6 months was associated with methylation in the *SNX25* and *LINC00840* genes, and exclusive breastfeeding for > 3 months was associated with methylation in the *FDFT1* gene at 10 years of age, but not at 18 years [18]. Odintsova et al. found that methylation at CpG sites in *ZNF232*, *MUCL1*, *DSCR3* and *ATG10* genes was associated with ever breastfeeding, but not with breastfeeding duration [19]. Finally, Hartwig et al. found that having ever been breastfed was associated with DNA methylation in the cg11414913 CpG at ages 7 and 15–17 years, but not at birth [20]. These studies were limited to examining breastfeeding as a measure of infant diet and have not looked at other aspects of the infant diet. This could be due to the high cost and challenge of gathering and compiling quality data about infant diet and the long-term follow-up required to measure methylation later in childhood. Since breastfeeding has been shown to influence DNA methylation, it is likely that other aspects of infant diet may have an effect as well.

Because DNA methylation plays a large part in regulation for many genes, it is important to understand how infant diet changes DNA methylation across the genome. This study aims to address the gaps in knowledge on infant diet exposures and their effects on DNA methylation. We conducted an epigenome-wide association study (EWAS) with several infant diet exposures, such as breastfeeding duration and age at exposure to gluten-containing cereals, non-gluten-containing cereals, fruit, vegetables, and meat. In addition, we aimed to assess if the associations found in infancy persist into childhood and if they were present at birth, to assess if factors other than infant diet exposures were at play.

## 2. Materials and Methods

### 2.1. Study Population

The study population was selected from the Diabetes Autoimmunity Study in the Young (DAISY), a prospective cohort of 2547 children at moderate to high risk for type 1 diabetes (T1D), which has been described previously [21,22]. In brief, this study recruited (1) children born in St. Joseph’s hospital whose umbilical cord blood was screened positive for T1D susceptibility alleles in the HLA-DR region or (2) children who had a first-degree relative with T1D. DAISY enrolled participants from 1993 through 2006 and is following them for the onset of autoimmunity and T1D. The Colorado Multiple Institutional Review Board approved all DAISY study protocols (COMIRB 92-080). Informed consent and assent, if appropriate, were obtained from the parents/legal guardians of all children prior to participation in any research related activities. Infant diet exposures were measured with a questionnaire in which the parents were asked to report their infant’s dietary exposures regarding the previous 3 months at 3, 6, 9, 12, and 15 months of age of their infant. The questionnaire included questions regarding breastfeeding duration and type and timing of complementary food and beverage introduction. Study visits (for the collection of blood samples) were conducted at 9, 15, and 24 months and annually thereafter to determine the presence of islet autoantibodies in serum.

**Figure 1:**
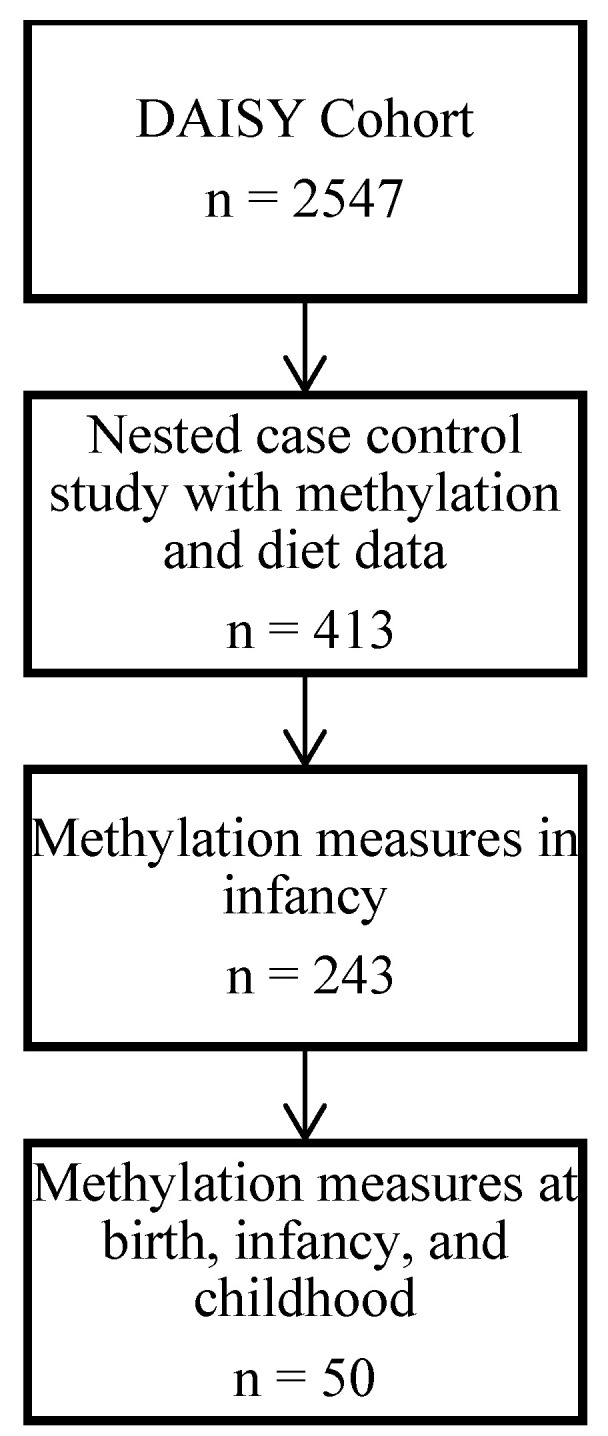
Flow chart of study population selection.

DAISY conducted a nested case–control study of 413 autoantibody-positive and autoantibody-negative children in whom DNA methylation in blood was measured at multiple timepoints throughout childhood. For our primary analysis, we selected the 243 children that had methylation measured during infancy (i.e., between 9 and 15 months of age) (Figure 1). We then investigated (1) whether the methylation differences persisted into childhood, and (2) whether the methylation differences detected in infancy pre-dated the infant diet exposures by examining cord blood methylation as a ‘negative control’ [23], with the reasoning that if the associations were present at birth, prenatal exposures or inherited differences related to infant diet behaviors were responsible rather than the dietary exposures themselves. To do this, we selected a subsample of 50 children that had methylation data at birth, in infancy and in childhood.

### 2.2. Measurement of DNA Methylation

DNA methylation was profiled in peripheral whole blood using the Infinium Human Methylation 450K Beadchip (Illumina, San Diego, CA, USA, “450 K”) or the Infinium Human Methylation EPIC Beadchip (“EPIC”). Children were randomly assigned to either the 450 K group (which included duplicate samples for quality control) or the EPIC group (which included replicates from the 450 K set for quality control). All visits of each individual were included on the same chip and in the same run. Both sets of data underwent identical pre-processing using the SeSAMe pipeline [24], and measurement platform (450 K or EPIC) was included as a covariate in all statistical models to account for technological batch effects. Johnson and colleagues performed quality control and removed poor quality samples and probes (see Johnson et al. for details) [25,26]. This resulted in 199,243 quality DNA methylation probes that were measured on both the 450 K and EPIC platforms. Normalized M values were used in all statistical analyses.

### 2.3. Infant Diet Variables

This study was designed as a prospective cohort to assess the association between infant diet variables and DNA methylation. We assessed six infant diet variables: breastfeeding duration (in months), exclusive breastfeeding duration (in months), and age at introduction to gluten-containing cereals (wheat, barley, rye), non-gluten-containing cereals (rice, oat), fruit (not including fruit juice), vegetables, and meat. The age at introduction variables were categorized using American Association of Pediatrics (AAP) guidelines regarding complementary feeding and associations previously found in DAISY [9] into three categories—introduction prior to 4 months of age, introduction from 4 to 5 months of age, and introduction at 6 or more (≥6) months of age. The 4–5 months of age category was used as the referent group. The age at introduction to meat and gluten-containing cereals variables were dichotomized into <6 months and ≥6 months (as the referent group) due to small samples sizes in the <4 months age category.

### 2.4. Statistical Analyses

We performed an EWAS to detect novel methylation sites associated with infant diet exposures at 9–15 months of age (infancy) in 243 children. In addition, we selected 17 CpG sites that had been significantly associated with breastfeeding in the previous literature and tested whether these CpG sites were associated with the infant diet variables in infancy in our cohort. We were unable to determine the actual CpG sites that were significant in the Pauwels et al. 2019 paper, as these were listed as ‘CpG2′and ‘CpG3′. Upon request, the authors provided the locations of these two regions and from these we selected 2 CpGs (for CpG2) and 5 CpGs (for CpG3) that were in our dataset and that were within 1000 kb at either end of the region as candidates for this analysis. The previous publications and the selected CpGs from each include Pauwels et al. 2019 [16] (cg00666422, cg12782180, cg13381984, cg14204281, cg19594666, cg24341498, cg26814075); Odintsova et al., 2019 [19] (cg03995300, cg11287055, cg16387046, cg16704958, cg27284194); Sherwood et al. 2019 [17] (cg03084214, cg23753947); Sherwood et al., 2020 [18] (cg04957663, cg14723566); and Hartwig et al. 2020 [20] (cg11414913).

We estimated the proportion of CD4+T cells, CD8+T cells, B cells, natural killer cells, granulocytes, and monocytes in each sample using the whole blood reference set [27,28]. We used multiple linear regression with infant diet variables as the exposure and DNA methylation as the outcome. We adjusted for age at methylation measure, sex, race/ethnicity (non-Hispanic white (NHW) vs. other), estimated cell proportions and methylation platform (450 K or EPIC) in all models. We assessed gestational age category (pre-term, term, post-term, based on maternal report) and birth weight as potential confounders, and they did not meet the classical definition of confounding and were therefore not included in the final models. For the EWAS, we used a false discovery rate (FDR) of <0.1 to determine significant associations. For the candidate CpGs from the previous literature, we used a nominal *p*-value of <0.05 to determine significance.

### 2.5. Analysis of Methylation Associations in Childhood and at Birth

The CpG sites that were associated with infant diet in infancy (9–15 months of age) were then analyzed in a subset of 50 children with methylation data at birth, in infancy and in childhood to examine if the associations seen in infancy were also present in childhood at 6–9 years of age, and if the associations present in infancy were present prior to the infant diet exposures. We focused on complete data (i.e., having all timepoints) so that the sample size was equivalent to avoid a power imbalance that would complicate interpretation of the results. We calculated a Bonferroni *p*-value cut-off of 0.0083, based on the 6 EWAS-significant CpGs that were tested in these analyses.

## 3. Results

Our primary study population consisted of 243 children with DNA methylation measures in infancy (at 9–15 months of age), of whom 47% were female, and 79.4% were non-Hispanic white (NHW) (Table 1). The population of 50 children with birth, infancy, and childhood methylation data was 50% female and 78% NHW. The distributions of infant diet variables were similar across the two analysis populations (Table 2).

### 3.1. Methylation in Infancy

**Figure 2:**
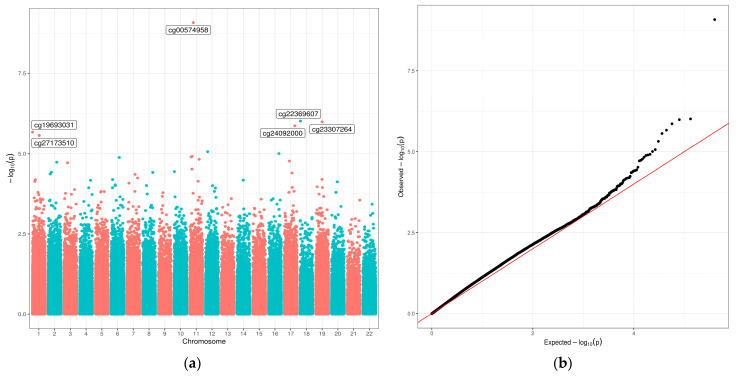
Manhattan plot (**a**) and Q-Q plot (**b**) for the epigenome-wide association study (EWAS) of breastfeeding duration in infancy (9–15 months). Breastfeeding duration was a continuous variable, in months. Models were adjusted for age at methylation measure, sex, race/ethnicity, cell composition, and platform. In the Manhattan plot (**a**), given names of six CpG sites indicate those that were associated with breastfeeding duration at FDR < 0.10.

We first conducted an EWAS of the infant diet variables and methylation during infancy (9–15 months). We identified six sites (cg00574958, cg19693031, cg22369607, cg23307264, cg24092000, and cg27173510) that were associated with breastfeeding duration months (FDR < 0.10) (Figure 2 and Table 3). The quantile–quantile (Q-Q) plot for breastfeeding duration shows no strong indication of genome-wide inflation (genomic inflation factor of 1.1) [29]. None of the other infant diet variables were associated with methylation in infancy (FDR ≥ 0.10).

### 3.2. Confirmation of Associations from Previous Literature

Of the 17 selected CpG sites previously found to be associated with breastfeeding or breastfeeding duration, DNA methylation at 4 CpG sites, near the Leptin gene (*LEP*), were associated with an infant diet variable in DAISY infants at 9–15 months. Higher methylation at cg13381984 and cg26814075 in infancy was significantly associated with shorter breastfeeding duration (*p* < 0.05); and higher methylation at cg00666422 and cg23752947 was associated with introduction to meat before 6 months of age (*p* < 0.05) (Table 3). No other associations with the previous candidate sites were detected.

### 3.3. Methylation in Childhood

Next, we examined whether the 10 associations between infant diet and methylation in infancy were present in childhood (6–9 years) in 50 children with methylation measures available at both timepoints (Table 4). We present results from both the infancy and childhood timepoints in this subset of 50 children for consistency and comparison. DNA methylation at 4 CpGs (cg00574958, cg22369607, cg24092000 and cg27173510) was significantly associated with breastfeeding duration at the childhood timepoint with a similar direction of effect as that seen in the original 243 children as well as in the 50 children in the overlapping subset at the infancy timepoint. None of the associations between breastfeeding duration or meat introduction and DNA methylation in *LEP* in infancy were present in childhood, nor were they present in infancy in this subsample of 50 children.

### 3.4. Methylation at Birth

Finally, we tested the 10 CpG sites in cord blood samples to determine whether the associations between infant diet and DNA methylation in infancy were present at birth (i.e., prior to the infant diet exposures) (Table 4). DNA methylation at three CpGs (cg22369607, cg24092000, and cg27173510) was significantly associated with breastfeeding duration at the birth timepoint with a similar direction of effect as that seen in the original 243 children as well as in the 50 children in the overlapping subset at the infancy timepoint. DNA methylation at cg26814075 at birth was marginally associated with breastfeeding duration with a similar direction of effect as that seen in the original infancy analysis. Neither of the two CpGs associated with age at introduction to meat in the original infancy analysis were associated at the birth timepoint.

## 4. Discussion

Compelling evidence of an effect of infant diet exposures on the epigenome is the presence of an association in infancy and childhood, and an absence of an association at birth. In our EWAS, we found that longer breastfeeding duration is associated with lower peripheral blood methylation at cg00574958 in *CPT1A* in infancy (9–15 months) and childhood (6–9 years), but not at birth.

*CPT1A* encodes the key enzyme (carnitine palmitoyltransferase 1A) in the carnitine-dependent multistep process that breaks down (metabolizes) fats and converts them into energy. Methylation at cg00574958 in *CPT1A* is associated with type 2 diabetes (T2D) [30,31], triglyceride levels [32], and blood pressure [33]. The expression of *CPT1A* in obese and overweight children is higher than that in normal weight children [34], and breastfeeding is associated with a decrease in *CPT1A* expression [35]. This, coupled with our findings, suggests that the long-term metabolic effects of breastfeeding may be through changes in methylation and expression of *CPT1A*.

Several studies have reported an inverse association between the duration of breastfeeding and methylation of CpG sites within *LEP* [16,17,36]. *LEP* encodes the hormone leptin, which is important in the regulation of energy intake by controlling satiety. We selected seven sites in *LEP* as significant candidates from the previous literature, and found that breastfeeding duration was inversely associated with methylation at cg13381984 and cg26814075 within *LEP* in infancy, and also that introduction to meat before 6 months was associated with increased methylation at cg00666422 and cg23753947 in *LEP* during infancy. While no other study has examined meat introduction with regard to *LEP*, the hypothesis would be that increased methylation would lead to decreased expression of *LEP*, thus increasing hunger leading to increased obesity and other metabolic disease [37]. These associations did not persist into childhood in our subsample of 50 children, but this may have been due to low power for cg13381984, cg26814075 and cg00666422, since the beta estimates were similar or only slightly attenuated in childhood, and even the infancy methylation associations became nonsignificant when tested in this small subsample.

Our study provides suggestive evidence of association between breastfeeding duration and methylation in infancy at cg19693031 (*TXNIP*), and cg23307264 (*KHSRP*) in infancy but not at birth. These associations were not present in childhood suggesting that the effect of infant diet did not persist. It is not clear whether these associations are merely false positives or truly transient effects. For *TXNIP* and *KHSRP*, it is unlikely that this lack of persistence is due to low power since the estimates in childhood were opposite to those in infancy. While methylation in *TXNIP* has been associated with type 2 diabetes [30,31], it has not been associated with breastfeeding or other infant diet exposures. Similarly, *KHSRP* has not been associated with infant diet exposures.

We tested methylation in cord blood to determine whether associations found in infancy were present at birth before any infant diet exposure. Methylation at cg22369607 (*AP001525.1*), cg24092000 (*TBCD*), and cg27173510 (*PGBD5*) was significantly associated with breastfeeding duration in both infancy and childhood, but also at birth. This could indicate prenatal exposures that are correlated with the mother’s decision to breastfeed longer that lead to methylation changes. An alternative explanation may be that the child inherited methylation marks associated with the breastmilk exposure of the mother, and that this exposure is associated with the decision of the mother to breastfeed her child. Further studies are needed to elucidate these potential explanations.

Limitations of this study were the small sample sizes available for the childhood and birth analyses. Moreover, the population was largely non-Hispanic white and at increased risk for type 1 diabetes, which affects the generalizability of the study results. Additionally, this study analyzed peripheral blood, which may not represent tissue-specific methylation, although we have adjusted for estimated cell proportions in all analyses.

## 5. Conclusions

This study has provided further evidence to support a role for infant diet in shaping a child’s epigenome. Our findings point to *CPT1A* as a candidate gene influenced by breastfeeding for further investigation. Future research should include large cohorts or consortia [38] to more accurately capture persistence of methylation associated with infant diet, and studies that include a more diverse population. Other areas of study include testing additional epigenetic mechanisms, such as histone modification, to determine if these are associated with infant diet.

## Figures and Tables

**Table 1:** Demographic and perinatal characteristics of the children that had methylation data at the different timepoints.

	Children with Methylation Data at the Infancy Timepoint (*n* = 243)	Children with Methylation Data at Birth, Infancy, and Childhood (*n* = 50)
Demographic Factors	N(%) unless otherwise specified	N(%) unless otherwise specified
Maternal age (years) at birth, mean (SD) ^a^	30.1 (5.2)	30.1 (4.7)
Maternal education		
>12 years	194 (80.8%)	42 (84.0%)
≤12 years		
Sex (Female)		
Female	114 (46.9%)	25 (50.0%)
Male		
Race/Ethnicity		
Non-Hispanic White	193 (79.4%)	39 (78.0%)
Other		
Perinatal Factors		
Birth weight (g), mean (SD) ^b^	3336.7 (516.0)	3380.3 (487.3)
Birth Delivery type		
Uncomplicated vaginal	146 (60.8%)	35 (70.0%)
Complicated vaginal	36 (15.0%)	8 (16.0%)
Cesarean section	58 (24.2%)	7 (14.0%)
Gestational Age Category ^c^		
Pre-term	42 (17.7%)	9 (18.0%)
Term	173 (72.7%)	32 (64.0%)
Post-term	23 (9.7%)	9 (18.0%)

^a^ Maternal age was missing for one child in the infancy analysis. ^b^ Birth weight was missing for four children in the infancy analysis. ^c^ Gestational age category was missing for five children in the infancy analysis.

**Table 2:** Infant diet exposures of the children that had methylation data at the different timepoints.

Exposure	Age at Introduction	Children with Methylation Data at the Infancy Timepoint (*n* = 243)	Children with Methylation Data at Birth, Infancy, and Childhood (*n* = 50)
Exclusive breastfeeding duration in months, mean (SD) ^a^	n.a.	1.9 (2.0)	2.0 (2.1)
Breastfeeding duration in months, mean (SD)	n.a.	7.3 (7.2)	9.0 (9.4)
		N (%)	N (%)
Age introduced to gluten-containing cereals (wheat/barley/rye)	<6 months	90 (37.0%)	14 (28.0%)
	≥6 months	153 (63.0%)	36 (72.0%)
Age introduced to non-gluten-containing cereals (rice/oat)	<4 months	91 (37.5%)	15 (30.0%)
	4–5 months	129 (53.1%)	28 (56.0%)
	≥6 months	23 (9.5%)	7 (14.0%)
Age introduced to fruit, excluding fruit juice	<4 months	34 (14.0%)	3 (6.0%)
	4–5 months	136 (56.0%)	30 (60.0%)
	≥6 months	73 (30.0%)	17 (34.0%)
Age introduced to vegetables	<4 months	22 (9.1%)	4 (8.0%)
	4–5 months	142 (58.4%)	27 (54.0%)
	≥6 months	79 (32.5%)	19 (38.0%
Age introduced to meat	<6 months	34 (14.0%)	4 (8.0%)
	≥6 months	209 (86.0%)	46 (92.0%)

^a^ Exclusive breastfeeding missing for 23 in the infancy timepoint and 5 in the birth, infancy, and childhood analysis. N, number; n.a., not applicable.

**Table 3:** Significant associations between infant diet and DNA methylation in infancy (9–15 months) in DAISY infants (*n* = 243) from an epigenome-wide association study (EWAS) and analysis of candidate CpGs from the previous literature.

		Position	Nearest Gene	Gene Region	Breastfeeding Duration (Months, Continuous)		Age at Introduction to Meat	
cgID	Chr				Beta Estimate	Nominal *p*-Value	Beta Estimate	Nominal *p*-Value
Significant (FDR < 0.10) CpGs from the Discovery EWAS								
cg00574958	11	68607622	*CPT1A*	5′UTR	−0.01741	8.34 × 10^−10^		
cg19693031	1	145441552	*TXNIP*	3′UTR	−0.01574	2.16 × 10^−6^		
cg22369607	18	13821885	AP001525.1 (miRNA)		−0.01507	9.76 × 10^−7^		
cg23307264	19	6424217	*KHSRP*	Body	0.025026	1.03 × 10^−6^		
cg24092000	17	80839375	*TBCD*	Body	−0.02497	1.37 × 10^−6^		
cg27173510	1	230468168	*PGBD5*	Body (in enhancer)	−0.01134	2.74 × 10^−6^		
Significant (*p* < 0.05) CpGs of Candidates from the Previous Literature								
cg13381984	7	127881344	*LEP*	1st exon, 5′UTR	−0.006	0.0121		
cg26814075	7	127881298	*LEP*	TSS200	−0.005	0.0323		
cg23753947	7	127889701	*LEP*	5′UTR			<6 m: 0.088 ≥6 m: ref	0.0482
cg00666422	7	127881440	*LEP*	5′UTR			<6 m: 0.095 ≥6 m: ref	0.0417

Models adjusted for age at methylation measure, sex, race/ethnicity, cell composition, and platform. Infant diet exposures examined include breastfeeding duration, exclusive breastfeeding duration and age at introduction of non-gluten-containing cereals (rice, oat), gluten-containing cereals (wheat, barley, rye), fruit (not including juice), vegetables, and meat. Only those showing a significant association with methylation at a CpG site are displayed in the table. Shaded grey cells indicate no association (FDR ≥ 0.1 for EWAS and nominal *p* ≥ 0.05 for candidate CpG analysis). TSS200: 0–200 bases upstream of the transcriptional start site (TSS).

**Table 4:** Examination of whether the significant infant diet and DNA methylation associations in infancy are present in childhood and/or at birth in a subsample of 50 children with methylation data at birth, infancy and childhood.

		Breastfeeding Duration (Months, Continuous)			Age at Introduction to Meat		
		Birth	Infancy	Childhood	Birth	Infancy	Childhood
cgID	Nearest Gene	Beta Estimate (*p*-Value)	Beta Estimate (*p*-Value)	Beta Estimate (*p*-Value)	Beta Estimate (*p*-Value)	Beta Estimate (*p*-Value)	Beta Estimate (*p*-Value)
Significant CpGs from the Discovery EWAS							
cg00574958	CPT1A	−0.002 (0.6062)	**−0.020 (2.35 × 10^−6^)**	**−0.015 (0.0011)**			
cg19693031	TXNIP	0.001 (0.8688)	−0.013 (0.0111)	0.003 (0.5905)			
cg22369607	AP001525.1	**−0.029 (0.0028)**	**−0.037 (9.58 × 10^−5^)**	**−0.025 (0.0031)**			
cg23307264	KHSRP	−0.003 (0.7816)	**0.073 (4.88 × 10^−5^)**	−0.010 (0.5385)			
cg24092000	TBCD	**−0.046 (0.0064)**	**−0.068 (1.18 × 10^−5^)**	**−0.064 (0.0001)**			
cg27173510	PGBD5	**−0.023 (0.0010)**	**−0.027 (0.0002)**	**−0.020 (0.0051)**			
Significant CpGs of Candidates from the Previous Literature							
cg13381984	LEP	−0.005 (0.3665)	−0.003 (0.4189)	−0.003 (0.5692)			
cg26814075	LEP	−0.009 (0.0732)	−0.006 (0.1424)	−0.003 (0.5622)			
cg23753947	LEP				<6 m: −0.335 (0.1119) ≥ 6 m: ref	<6 m: 0.065 (0.6018) ≥6 m: ref	<6 m: −0.158 (0.1963) ≥6 m: ref
cg00666422	LEP				<6 m: 0.190 (0.2065) ≥6 m: ref	<6 m: 0.248 (0.0685) ≥6 m: ref	<6 m: 0.218 (0.1324) ≥6 m: ref

Models adjusted for age at methylation measure, sex, race/ethnicity, cell composition, and platform. Shaded grey cells indicate associations that were not tested because they were not significant in the EWAS nor candidate CpG analysis in infancy. Nominal *p*-values are presented in the table. Those less than the Bonferroni *p*-value cut-off of 0.0083 are indicated by bolding.

## Data Availability

The data presented in this study are available on request from the corresponding author. The data are not publicly available because it contains protected health information.

## References

[1] Yan J., Liu L., Zhu Y., Huang G., Wang P.P. (2014). The association between breastfeeding and childhood obesity: A meta-analysis. BMC Public Health.

[2] Horta B.L., de Lima N.P. (2019). Breastfeeding and Type 2 Diabetes: Systematic Review and Meta-Analysis. Curr. Diab. Rep..

[3] Gungor D., Nadaud P., LaPergola C.C., Dreibelbis C., Wong Y.P., Terry N., Abrams S.A., Beker L., Jacobovits T., Jarvinen K.M. (2019). Infant milk-feeding practices and diabetes outcomes in offspring: A systematic review. Am. J. Clin. Nutr..

[4] Butte N.F., Lopez-Alarson M.G., Garza C. (2002). Expert Consultation on the Optimal Duration of Exclusive Breastfeeding. Nutrient Adequacy of Exclusive Breastfeeding for the Term Infant during the First Six Months of Life.

[5] Logan K., Perkin M.R., Marrs T., Radulovic S., Craven J., Flohr C., Bahnson H.T., Lack G. (2020). Early Gluten Introduction and Celiac Disease in the EAT Study: A Prespecified Analysis of the EAT Randomized Clinical Trial. JAMA Pediatr..

[6] Norris J.M., Barriga K., Hoffenberg E.J., Taki I., Miao D., Haas J.E., Emery L.M., Sokol R.J., Erlich H.A., Eisenbarth G.S. (2005). Risk of celiac disease autoimmunity and timing of gluten introduction in the diet of infants at increased risk of disease. JAMA.

[7] Lionetti E., Castellaneta S., Francavilla R., Pulvirenti A., Tonutti E., Amarri S., Barbato M., Barbera C., Barera G., Bellantoni A. (2014). Introduction of gluten, HLA status, and the risk of celiac disease in children. N. Engl. J. Med..

[8] Vriezinga S.L., Auricchio R., Bravi E., Castillejo G., Chmielewska A., Crespo Escobar P., Kolacek S., Koletzko S., Korponay-Szabo I.R., Mummert E. (2014). Randomized feeding intervention in infants at high risk for celiac disease. N. Engl. J. Med..

[9] Frederiksen B., Kroehl M., Lamb M.M., Seifert J., Barriga K., Eisenbarth G.S., Rewers M., Norris J.M. (2013). Infant exposures and development of type 1 diabetes mellitus: The Diabetes Autoimmunity Study in the Young (DAISY). JAMA Pediatr..

[10] Norris J.M., Barriga K., Klingensmith G., Hoffman M., Eisenbarth G.S., Erlich H.A., Rewers M. (2003). Timing of initial cereal exposure in infancy and risk of islet autoimmunity. JAMA.

[11] Virtanen S.M., Kenward M.G., Erkkola M., Kautiainen S., Kronberg-Kippila C., Hakulinen T., Ahonen S., Uusitalo L., Niinisto S., Veijola R. (2006). Age at introduction of new foods and advanced beta cell autoimmunity in young children with HLA-conferred susceptibility to type 1 diabetes. Diabetologia.

[12] Virtanen S.M., Takkinen H.M., Nevalainen J., Kronberg-Kippila C., Salmenhaara M., Uusitalo L., Kenward M.G., Erkkola M., Veijola R., Simell O. (2011). Early introduction of root vegetables in infancy associated with advanced ss-cell autoimmunity in young children with human leukocyte antigen-conferred susceptibility to Type 1 diabetes. Diabet. Med..

[13] Canani R.B., Costanzo M.D., Leone L., Bedogni G., Brambilla P., Cianfarani S., Nobili V., Pietrobelli A., Agostoni C. (2011). Epigenetic mechanisms elicited by nutrition in early life. Nutr. Res. Rev..

[14] Moore L.D., Le T., Fan G. (2013). DNA methylation and its basic function. Neuropsychopharmacology.

[15] Hartwig F.P., Loret de Mola C., Davies N.M., Victora C.G., Relton C.L. (2017). Breastfeeding effects on DNA methylation in the offspring: A systematic literature review. PLoS ONE.

[16] Pauwels S., Symons L., Vanautgaerden E.L., Ghosh M., Duca R.C., Bekaert B., Freson K., Huybrechts I., Langie S.A.S., Koppen G. (2019). The Influence of the Duration of Breastfeeding on the Infant’s Metabolic Epigenome. Nutrients.

[17] Sherwood W.B., Bion V., Lockett G.A., Ziyab A.H., Soto-Ramirez N., Mukherjee N., Kurukulaaratchy R.J., Ewart S., Zhang H., Arshad S.H. (2019). Duration of breastfeeding is associated with leptin (*LEP*) DNA methylation profiles and BMI in 10-year-old children. Clin. Epigenet..

[18] Sherwood W.B., Kothalawala D.M., Kadalayil L., Ewart S., Zhang H., Karmaus W., Arshad S.H., Holloway J.W., Rezwan F.I. (2020). Epigenome-Wide Association Study Reveals Duration of Breastfeeding Is Associated with Epigenetic Differences in Children. Int. J. Environ. Res. Public Health.

[19] Odintsova V.V., Hagenbeek F.A., Suderman M., Caramaschi D., van Beijsterveldt C.E.M., Kallsen N.A., Ehli E.A., Davies G.E., Sukhikh G.T., Fanos V. (2019). DNA Methylation Signatures of Breastfeeding in Buccal Cells Collected in Mid-Childhood. Nutrients.

[20] Hartwig F.P., Davey Smith G., Simpkin A.J., Victora C.G., Relton C.L., Caramaschi D. (2020). Association between Breastfeeding and DNA Methylation over the Life Course: Findings from the Avon Longitudinal Study of Parents and Children (ALSPAC). Nutrients.

[21] Rewers M., Bugawan T.L., Norris J.M., Blair A., Beaty B., Hoffman M., McDuffie R.S., Hamman R.F., Klingensmith G., Eisenbarth G.S. (1996). Newborn screening for HLA markers associated with IDDM: Diabetes autoimmunity study in the young (DAISY). Diabetologia.

[22] Rewers M., Norris J.M., Eisenbarth G.S., Erlich H.A., Beaty B., Klingensmith G., Hoffman M., Yu L., Bugawan T.L., Blair A. (1996). Beta-cell autoantibodies in infants and toddlers without IDDM relatives: Diabetes autoimmunity study in the young (DAISY). J. Autoimmun..

[23] Smith G.D. (2008). Assessing intrauterine influences on offspring health outcomes: Can epidemiological studies yield robust findings?. Basic Clin. Pharmacol. Toxicol..

[24] Zhou W., Triche T.J., Laird P.W., Shen H. (2018). SeSAMe: Reducing artifactual detection of DNA methylation by Infinium BeadChips in genomic deletions. Nucleic Acids Res..

[25] Johnson R.K., Vanderlinden L.A., Dong F., Carry P.M., Seifert J., Waugh K., Shorrosh H., Fingerlin T., Frohnert B.I., Yang I.V. (2020). Longitudinal DNA methylation differences precede type 1 diabetes. Sci. Rep..

[26] Vanderlinden L.A., Johnson R.K., Carry P.M., Dong F., DeMeo D.L., Yang I.V., Norris J.M., Kechris K. (2021). An effective processing pipeline for harmonizing DNA methylation data from Illumina’s 450K and EPIC platforms for epidemiological studies. BMC Res. Notes.

[27] Houseman E.A., Accomando W.P., Koestler D.C., Christensen B.C., Marsit C.J., Nelson H.H., Wiencke J.K., Kelsey K.T. (2012). DNA methylation arrays as surrogate measures of cell mixture distribution. BMC Bioinform..

[28] Jaffe A.E., Irizarry R.A. (2014). Accounting for cellular heterogeneity is critical in epigenome-wide association studies. Genome Biol..

[29] van Iterson M., van Zwet E.W., Consortium B., Heijmans B.T. (2017). Controlling bias and inflation in epigenome- and transcriptome-wide association studies using the empirical null distribution. Genome Biol..

[30] Juvinao-Quintero D.L., Marioni R.E., Ochoa-Rosales C., Russ T.C., Deary I.J., van Meurs J.B.J., Voortman T., Hivert M.F., Sharp G.C., Relton C.L. (2021). DNA methylation of blood cells is associated with prevalent type 2 diabetes in a meta-analysis of four European cohorts. Clin. Epigenet..

[31] Kim H., Bae J.H., Park K.S., Sung J., Kwak S.H. (2021). DNA Methylation Changes Associated with Type 2 Diabetes and Diabetic Kidney Disease in an East Asian Population. J. Clin. Endocrinol. Metab..

[32] Jhun M.A., Mendelson M., Wilson R., Gondalia R., Joehanes R., Salfati E., Zhao X., Braun K.V.E., Do A.N., Hedman A.K. (2021). A multi-ethnic epigenome-wide association study of leukocyte DNA methylation and blood lipids. Nat. Commun..

[33] Richard M.A., Huan T., Ligthart S., Gondalia R., Jhun M.A., Brody J.A., Irvin M.R., Marioni R., Shen J., Tsai P.C. (2017). DNA Methylation Analysis Identifies Loci for Blood Pressure Regulation. Am. J. Hum. Genet..

[34] Sanchez J., Priego T., Pico C., Ahrens W., De Henauw S., Fraterman A., Marild S., Molnar D., Moreno L.A., Peplies J. (2012). Blood cells as a source of transcriptional biomarkers of childhood obesity and its related metabolic alterations: Results of the IDEFICS study. J. Clin. Endocrinol. Metab..

[35] Cheshmeh S., Nachvak S.M., Rezvani N., Saber A. (2020). Effects of Breastfeeding and Formula Feeding on the Expression Level of FTO, *CPT1A* and PPAR-alpha Genes in Healthy Infants. Diabetes Metab. Syndr. Obes..

[36] Obermann-Borst S.A., Eilers P.H., Tobi E.W., de Jong F.H., Slagboom P.E., Heijmans B.T., Steegers-Theunissen R.P. (2013). Duration of breastfeeding and gender are associated with methylation of the *LEPTIN* gene in very young children. Pediatr. Res..

[37] Paracchini V., Pedotti P., Taioli E. (2005). Genetics of leptin and obesity: A HuGE review. Am. J. Epidemiol..

[38] Felix J.F., Joubert B.R., Baccarelli A.A., Sharp G.C., Almqvist C., Annesi-Maesano I., Arshad H., Baiz N., Bakermans-Kranenburg M.J., Bakulski K.M. (2018). Cohort Profile: Pregnancy And Childhood Epigenetics (PACE) Consortium. Int. J. Epidemiol..

